# The quality–coverage gap in antenatal care: toward better measurement of effective coverage

**DOI:** 10.9745/GHSP-D-13-00176

**Published:** 2014-04-08

**Authors:** Stephen Hodgins, Alexis D'Agostino

**Affiliations:** a Save the Children, Washington, DC, USA; b John Snow, Inc., Arlington, VA, USA

## Abstract

**Background:**

The proportion of pregnant women receiving 4 or more antenatal care visits (ANC 4+) is used prominently as a global benchmark indicator to track maternal health program performance. This has contributed to an inappropriate focus on the number of contacts rather than on the content and process of care. This paper presents analysis of specific elements of care received among women reporting 4 or more ANC visits.

**Methods:**

We conducted secondary analysis using Demographic and Health Survey data from 41 countries to determine coverage for specific elements of antenatal care. The analysis was conducted for: (1) women who delivered during the 2 years preceding the survey and who reported receiving 4 or more ANC visits, and (2) *all* women who delivered during the preceding 2 years. The specific ANC services that we assessed were: blood pressure measurement, tetanus toxoid vaccination, first ANC visit at less than 4 months gestation, urine testing, counseling about danger signs, HIV counseling and testing, iron–folate supplementation (≥ 90 days), and at least 2 doses of sulfadoxine/pyramethamine for malaria prevention. The difference between expected (100%) and actual coverage (the quality–coverage gap) was calculated for each service across the 41 surveys.

**Results:**

Coverage for specific elements of care among women reporting 4 or more ANC visits was generally low for most of the specific elements assessed. Blood pressure and tetanus toxoid performed best, with median quality–coverage gaps of 5% and 18%, respectively. The greatest gaps were for iron–folate supplementation (72%) and malaria prevention (86%). Coverage for specific interventions was generally much lower among all pregnant women (reflecting population effective coverage) than among only those who had received ANC 4+ visits. Although ANC 4+ and average coverage across the elements of care correlated relatively well (Pearson r^2^  =  0.56), most countries had lower average coverage for the content of care than for ANC 4+ (among all pregnant women).

**Conclusion:**

We argue for the adoption of a summary indicator that better reflects the content of antenatal care than does the current ANC 4+ indicator. We propose, as an alternative, the simple average of a set of ANC content indicators available through surveys and routine health information systems.

## Abstract (precis)

The proportion of pregnant women receiving 4 or more antenatal care (ANC) visits has no necessary relationship with the actual content of those visits. We propose a simple alternative to measure program performance that aggregates key services that are common across countries and measured in Demographic and Health Surveys, such as blood pressure measurement, tetanus toxoid vaccination, first ANC visit before 4 months gestation, urine testing, counseling about pregnancy danger signs, and iron–folate supplementation.

## INTRODUCTION

The **proportion of pregnant women receiving 4 or more antenatal care visits** (ANC 4+) has pride of place as a global benchmark indicator, standing in as a proxy for adequacy of antenatal care (ANC). It has been used as an indicator both for Millennium Development Goal 5 (improve maternal health)^1^ and for the United Nations Secretary General's Commission for Information and Accountability for Women's and Children's Health.^2^

In the late 1990s, José Villar led a multicountry study,^3^ under the auspices of the World Health Organization (WHO), comparing a more goal-oriented, abbreviated, 4-visit schedule with conventional ANC. Conventional ANC comprised about 12 visits (one visit each month during the first 6 months of pregnancy, once every 2–3 weeks for the next 2 months, and once a week thereafter until delivery). On most measures, there were no differences in maternal or perinatal outcomes. These findings have been the basis for adoption of the ANC 4+ indicator as a marker of receipt of adequate antenatal care.

Since that time, along with skilled birth attendance, ANC 4+ has been the most frequently used summary measure of maternal health program performance. This has had the unfortunate consequence of drawing the attention of program managers away from the *content and process of care* and toward mere *contact*. But content and process of care matter. As Bhutta and colleagues have documented in their comprehensive review,^4^ there is significant scope for improving health outcomes, even with a simple package of antenatal interventions that can be delivered by health auxiliaries consisting of:

- Tetanus toxoid
- Intermittent presumptive/preventive treatment of malaria
- Iron–folate and calcium supplementation
- Deworming
- Detection and treatment of preeclampsia, syphilis, and asymptomatic bacteriuria
- Counseling about essential newborn care practices (immediate and exclusive breastfeeding, clean delivery, and thermal protection) and care-seeking for institutional delivery and danger signs

Focusing on the proportion of pregnant women making at least 4 antenatal visits to measure program performance has drawn the attention away from the content of care to mere contact.

Clearly, it is not mere contact that results in better outcomes; it is the actual substance of care delivered. Using data from the Demographic and Health Surveys (DHS), this paper explores the extent to which the ANC 4+ indicator tells us anything useful about the substance of care and proposes an alternative indicator to measure program performance.

## METHODS

Recent DHS data from 41 countries were analyzed, retaining information on pregnancies during the preceding 2 years for which the mother reported receiving 4 or more ANC visits. From these data, we determined the proportion of survey respondents who reported receipt of 8 specific clinical preventive services:

- Blood pressure measurement
- Full protection against tetanus
- First antenatal visit at less than 4 months gestation
- Urine testing
- Counseling about danger signs
- HIV counseling and testing
- Iron–folate supplementation for at least 90 days
- At least 2 doses of sulfadoxine/pyramethamine (SP) for presumptive/preventive malaria treatment

Surveys retained for this analysis had to have values for at least 5 of these interventions of interest. Among the surveys retained, the main distinction in which data were included was the presence or absence of HIV- and malaria-related indicators. A “quality–coverage gap” was calculated for each of these services—across the 41 surveys—as the difference between expected (100%) and actual coverage.

We also present additional DHS analysis on coverage for this set of services using, as the denominator, *all* women having a birth in the 2 years preceding the survey (regardless of the number of ANC visits received). For each country survey, a simple mean was calculated across the set of retained antenatal indicators listed above as well as the proportion of women who reported receiving *all* the interventions.

The country surveys were conducted by MEASURE DHS, a project of the Bureau for Global Health at the U.S. Agency for International Development (USAID). All the datasets are available online at www.dhsprogram.com. Analysis was done using Stata 12.1. In line with DHS practice, women not providing a response or answering “do not know” to questions on services received were retained in the denominators for calculation of the indicators (that is, it was assumed that they did not receive those services).^5^ Results from each country were calculated using the weighting and sampling information and procedures specified in the DHS datasets and documentation.

## RESULTS

### Quality of Care Among Those Receiving 4+ Visits

The analysis presented in Table 1 can be considered as characterizing the quality of care received, among women who reported receiving 4 or more ANC visits. Colombia, the Dominican Republic, and Nepal performed well; *average* coverage across the indicators measured in those surveys was 83%–85% (a quality–coverage gap of 15%–17%). Although Nepal performed as well as the other 2 countries with regard to average coverage, a considerably smaller proportion of pregnant women in Nepal reported 4+ visits (53% versus 87% in Colombia and 96% in the Dominican Republic). Timor-Leste, Indonesia, and Lesotho were the median performers across the 41 countries, with average coverage across indicators of 58% (average quality–coverage gap of 42%). The poorest performing countries were the Democratic Republic of Congo and Burundi, with an average coverage across indicators of 32% and 36% (quality–coverage gaps of 68% and 64%, respectively).

**TABLE 1.** Receipt of Specific Services Among Pregnant Women With 4+ ANC Visits^a^ (%)

**Survey**	**ANC4+**	**ANC<4mo**	**IFA90+**	**TT2+**	**DSs**	**BP**	**Ur**	**HIV**	**SP2+**	**AVG**
Colombia 2010	**87**	82		62	83	100	98	85		**85**
Dominican Rep 2007	**96**	84	66	92	70	99	97	86		**85**
Nepal 2011	**53**	72	78	97	87	94	72			**83**
Maldives 2009	**87**	93	65	86	51	97	95			**81**
Honduras 2005–06	**79**	79	73	75	68	97	79			**78**
Rwanda 2010	**36**	73			74	88	43	96		**75**
Peru 2007–08	**88**	75	17	69	84	98	81			**71**
India 2005–06	**36**	80	32	93	34	89	85			**69**
Philippines 2008	**76**	61	35	80	75	96	60			**68**
Senegal 2010–11	**48**	78	68	75	48	98	88	39	47	**68**
Burkina Faso 2010	**33**	65	50	91	56	97	89	41	50	**67**
Ghana 2008	**76**	65	44	77	75	98	93	32	51	**67**
Bolivia 2008	**72**	76	10	66	70	98	77			**66**
Cambodia 2010	**64**	81	14	94	83	96	42	48		**65**
Guyana 2009	**77**	53	30	42	64	96	94	78		**65**
Haiti 2005–06	**51**	73	32	76	50	98	76	36		**63**
Pakistan 2006–07	**29**	70	29	84	33	92	70			**63**
Cameroon 2011	**59**	47	65	87	50	96	89	30	35	**62**
Swaziland 2006–07	**77**	27	30	82	54	98	91	53		**62**
Malawi 2010	**43**	23	27	90	81	85	31	88	61	**61**
Timor-Leste 2009–10	**54**	64	20	91	61	95	20			**58**
Indonesia 2007	**81**	84	31	56	43	95	42			**58**
Lesotho 2009	**66**	40	9	84	59	97	73	46		**58**
Namibia 2006–07	**70**	36	29	56	63	97	92	73	11	**57**
Benin 2006	**59**	61	61	74	42	99	93	20	4	**57**
Ethiopia 2011	**17**	40		81	29	83	56	49		**56**
Zambia 2007	**57**	26	41	83	75	79	21	39	74	**55**
Kenya 2008–09	**44**	26	3	83	53	89	76	83	20	**54**
Liberia 2007	**66**	72	13	86	40	87	52		14	**52**
Tanzania 2010	**39**	26	2	93	56	71	58	75	34	**52**
Guinea 2005	**46**	49	35	86	29	93	65		5	**52**
Zimbabwe 2010–11	**59**	25	5	64	66	88	60	82	10	**50**
Uganda 2011	**46**	33	6	90	57	66	28	78	31	**49**
Nigeria 2008	**44**	28	22	77	64	86	75	26	10	**48**
Congo (Brazzaville) 2005	**72**	55	17	54	40	95	95	8	3	**46**
Mali 2006	**36**	57	24	78	32	92	53	11	17	**46**
Sierra Leone 2008	**56**	39	15	87	60	87	42	12	14	**44**
Madagascar 2008–09	**46**	42	8	79	52	83	34	8	8	**39**
Niger 2006	**15**	46	25	57	28	90	46	5	13	**39**
Burundi 2010	**33**	39	7	91	40	50	12	46	1	**36**
Dem Rep Congo 2007	**47**	28	2	47	42	73	51	8	8	**32**
**Mean**	**57**	**55**	**30**	**79**	**58**	**91**	**67**	**49**	**25**	**60**

Abbreviations: ANC4+, 4 or more antenatal care visits; ANC<4mo, first antenatal care visit before 4 months gestation; BP, blood pressure; DSs, counseled on pregnancy danger signs; HIV, HIV counseling and testing; IFA, iron–folic acid supplementation for 90+ days; SP2+, at least 2 doses of sulfadoxine/pyramethamine for malaria prevention; TT2+, protected against tetanus; Ur, urine specimen taken.

AVG: Average coverage across the 8 interventions (or fewer, if specific intervention(s) not included in the survey). Country data are presented in order, from highest *average* coverage to lowest.

a Self-reported receipt of services among women delivering during the 2 years preceding the survey and reporting 4+ ANC visits.

As seen in the Figure, with the exception of blood pressure measurement, there were marked quality–coverage gaps for each of these elements of care for most countries, ranging from 18% to 86%. The greatest gap was for 2 commodity-dependent functions—iron–folate supplementation (72%) and presumptive/preventive treatment for malaria with SP (86%). (HIV testing and tetanus toxoid are also commodity-dependent, but supply is commonly managed under separate, vertical systems; iron–folate and SP provision normally does not benefit from such special logistical arrangements.)

**FIGURE.**
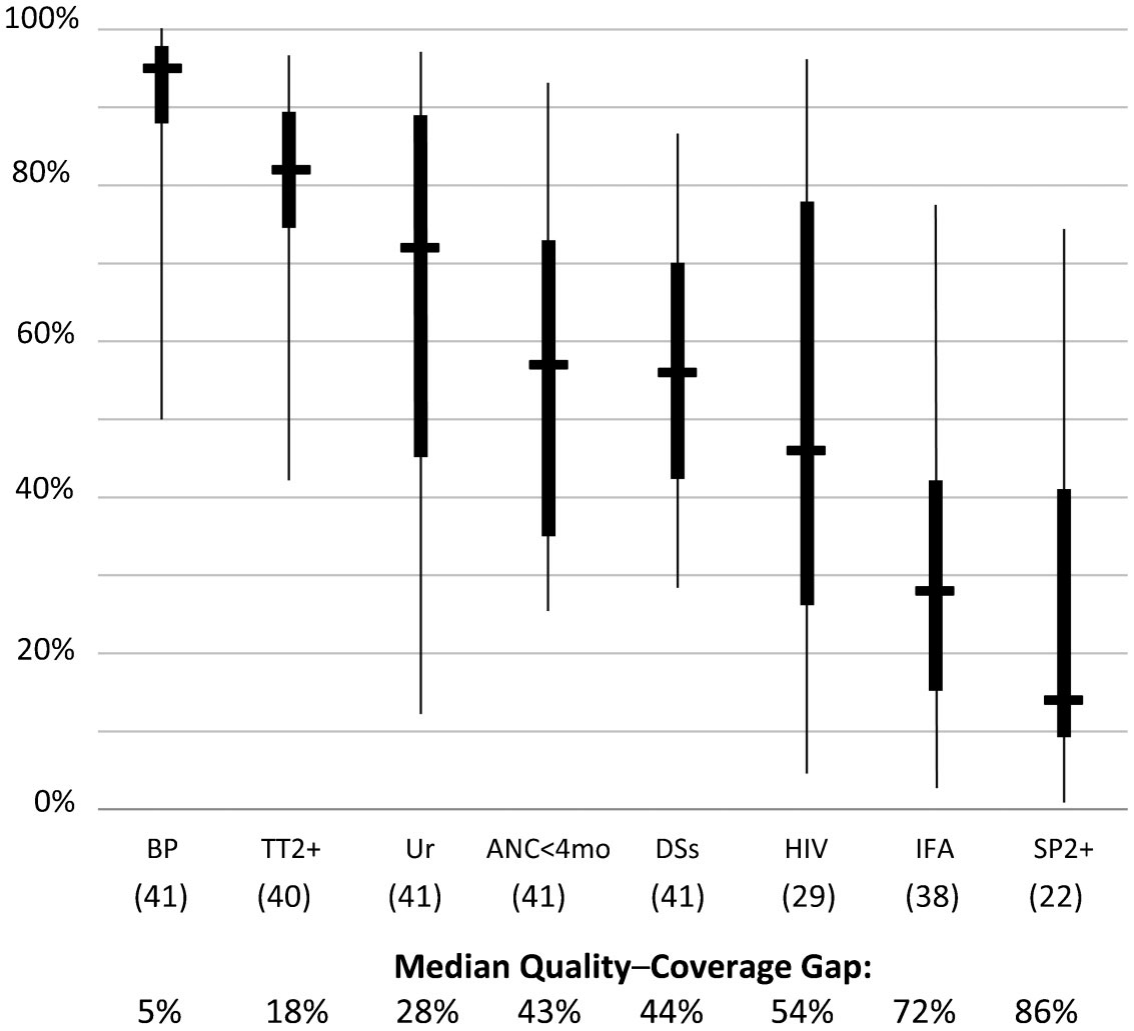
Coverage for Key ANC Services Among Pregnant Women With 4+ ANC Visits,a Across 41 Demographic and Health Surveys ^a^ Self-reported receipt of services among women delivering during the 2 years preceding the survey and reporting 4+ ANC visits. Abbreviations: ANC, antenatal care; ANC<4mo, first antenatal care visit before 4 months gestation; BP, blood pressure; DSs, counseled on pregnancy danger signs; HIV, HIV counseling and testing; IFA, iron–folic acid supplementation for 90+ days; SP2+, at least 2 doses of sulfadoxine/pyramethamine for malaria prevention; TT2+, protected against tetanus; Ur, urine specimen taken. The horizontal line in the middle of each solid box indicates the median; the top and bottom borders of the box mark the 75th and 25th percentiles, respectively. The “whiskers,” or lines, below and above the box mark the minimum and maximum values, respectively. Numbers in parentheses in the x-axis refer to the number of surveys providing data for that particular indicator.

The greatest quality–coverage gaps were for iron–folate supplementation and preventive treatment for malaria, both of which depend on reliable commodity supplies.

### Effective Coverage at Population Level

Whereas Table 1 presented intervention-specific coverage among those reporting 4 or more ANC visits (that is, those who are supposedly “covered” with respect to ANC services), Table 2 presents data calculated for *all* women delivering over the previous 2 years as the denominator, reflecting effective coverage at the population level. Specifically, mean coverage across *all* the antenatal indicators offers an alternative summary measure that could be considered for antenatal program performance.

**TABLE 2.** Receipt of Specific Services Among *All* Pregnancies^a^ (%)

**Survey**	**ANC4+**	**ANC<4mo**	**IFA90+**	**TT2+**	**DSs**	**BP**	**Ur**	**HIV**	**SP2+**	**AVG**	**ALL**
Dominican Rep 2007	**96**	81	65	91	69	99	96	86		**84**	34
Maldives 2009	**87**	90	64	86	51	97	95			**81**	28
Colombia 2010	**87**	74		59	79	96	94	80		**80**	34
Honduras 2005–06	**79**	65	61	71	60	89	68			**69**	10
Rwanda 2010	**36**	40		84	71	84	36	92		**68**	8
Peru 2007–08	**88**	68	15	66	78	93	75			**66**	7
Nepal 2011	**53**	50	52	84	66	76	51			**63**	22
Guyana 2009	**77**	46	29	42	62	94	91	71		**62**	4
Philippines 2008	**76**	50	28	74	67	89	52			**60**	10
Ghana 2008	**76**	54	36	70	67	94	86	27	46	**60**	5
Senegal 2010–11	**48**	58	56	70	42	93	80	30	40	**59**	3
Swaziland 2006–07	**77**	22	25	78	50	96	86	50		**58**	2
Burkina Faso 2010	**33**	40	34	86	50	93	81	31	39	**57**	1
Bolivia 2008	**72**	60	8	58	60	89	65			**57**	3
Cambodia 2010	**64**	63	10	88	73	84	34	38		**56**	2
Malawi 2010	**43**	12	15	87	78	82	27	85	55	**55**	1
Namibia 2006–07	**70**	30	24	56	58	92	88	68	11	**53**	0
Indonesia 2007	**81**	74	25	50	39	88	37			**52**	4
Lesotho 2009	**66**	30	6	75	49	88	63	42		**50**	1
Zambia 2007	**57**	18	28	79	70	75	19	39	66	**49**	1
Cameroon 2011	**59**	32	43	74	40	79	72	23	26	**49**	2
Haiti 2005–06	**51**	61	18	62	38	82	55	24		**49**	8
Timor-Leste 2009–10	**54**	43	12	78	48	82	15			**46**	1
Benin 2006	**59**	40	42	61	34	88	81	14	3	**45**	0
Tanzania 2010	**39**	14	1	91	50	64	47	68	27	**45**	0
Liberia 2007	**66**	59	11	77	38	79	46		0	**44**	2
Kenya 2008–09	**44**	14	1	72	40	77	61	70	15	**44**	0
Uganda 2011	**46**	20	3	85	49	55	21	72	27	**42**	0
Zimbabwe 2010–11	**59**	16	3	54	55	75	50	68	8	**41**	0
India 2005–06	**36**	43	15	76	19	48	44			**41**	4
Sierra Leone 2008	**56**	29	13	81	56	81	37	10	12	**40**	0
Congo (Brazzaville) 2005	**72**	42	13	45	34	82	82	7	0	**38**	0
Guinea 2005	**46**	32	19		22	72	47		4	**32**	0
Madagascar 2008–09	**46**	25	4	68	43	71	23	6	7	**31**	0
Burundi 2010	**33**	19	3	87	35	44	9	40	0	**30**	0
Nigeria 2008	**44**	16	12	48	38	53	46	15	6	**29**	0
Mali 2006	**36**	30	12	57	21	64	31	6	11	**29**	0
Dem Rep Congo 2007	**47**	18	1	37	33	62	42	6	7	**26**	0
Pakistan 2006–07	**29**	31	12	60	17	52	32	0	0	**25**	2
Ethiopia 2011	**17**	10		49	9	31	17	16		**22**	1
Niger 2006	**15**	13	7	23	12	41	18	1	0	**14**	0
**Mean**	**57**	**40**	**21**	**69**	**49**	**78**	**55**	**43**	**20**	**50**	

Abbreviations: ANC4+, 4 or more antenatal care visits; ANC<4mo, first antenatal care visit before 4 months gestation; BP, blood pressure; DSs, counseled on pregnancy danger signs; HIV, HIV counseling and testing; IFA, iron–folic acid supplementation for 90+ days; SP2+, at least 2 doses of sulfadoxine/pyramethamine for malaria prevention; TT2+, protected against tetanus; Ur, urine specimen taken.

AVG: Average coverage across the 8 interventions (or fewer, if specific intervention(s) not included in the survey). Country data are presented in order, from highest *average* coverage to lowest.

a Self-reported receipt of services among all women delivering during the 2 years preceding the survey.

The 2 tables (Table 1, reflecting ANC quality, and Table 2, reflecting population effective coverage) show somewhat similar rankings. For example, the top 7 performers are the same on these 2 measures. Most countries were underperformers—in the sense that average population effective coverage for actual content was lower than for ANC 4+. For only 8 of the 41 countries was average coverage higher than the proportion of women reporting 4 or more visits (Table 2). (This is reflected in the generally large quality–coverage gaps for individual interventions.)

Four of the 10 highest-performing countries, with respect to average coverage across the specific elements of care, also had ANC 4+ values greater than 85% (Dominican Republic, Maldives, Colombia, and Peru) (Table 2). On the other hand, 2 of these 10 countries had comparatively low ANC 4+ values: Rwanda (36%) and Nepal (53%). Very low average coverage was generally associated with low ANC 4+. However, there were several cases of relatively low coverage on specific antenatal content in countries with relatively high ANC 4+ (for example, Congo Brazzaville, with average coverage of 38% and ANC 4+ of 72%; Indonesia, with average coverage of 52% and ANC 4+ of 81%; and Namibia, with average coverage of 53% and ANC 4+ of 70%).

### Correlation Between Number of Visits and Care Received

Certainly, in general, the more ANC visits one has, the higher the likelihood of receiving specific elements of care. So, not surprisingly, ANC 4+ and mean coverage across the 8 elements of care correlate relatively well (Pearson r^2^ = 0.56). In other words, 56% of the variance in mean coverage is accounted for by the value of ANC 4+. The number of visits does matter, in the sense that each visit provides an opportunity for provision of needed care. Fewer visits means fewer opportunities.

Mean number of visits correlates similarly well (r^2^ = 0.53), and has the advantage that its use as an indicator would not (inappropriately) signal that any particular number of visits is automatically sufficient. Regardless of degree of association, whether with ANC 4+ or mean number of visits, as is evident in the data presented here, there is no necessary relationship with reliable delivery of the content of care.

### Receipt of the Full Set of Interventions

Among all pregnancies during the 2 years preceding the survey, the proportion of women who reported receiving *all* 8 services (or fewer, if a particular indicator was not included in the survey) was zero in over one-third of the surveys (15 of 41) (Table 2). In only 4 countries was the proportion 20% or higher (Dominican Republic, Maldives, Colombia, and Nepal). In Honduras and the Philippines, the proportion was 10%; in Rwanda and Haiti, 8%; and in Peru, 7%. In none of the other countries was it above 5%.

## DISCUSSION

As this analysis demonstrates, there are large quality–coverage gaps for most of the antenatal interventions assessed. Such gaps mean ineffective care, and ineffective care means missed opportunities to achieve better outcomes. Focusing on mere contact rather than on the content of care means that we have taken our eye off what really matters.

Most ANC services assessed had large quality–coverage gaps, reflecting ineffective care.

**ANC 1** (*any* ANC) and **ANC visit within the first 4 months of gestation** are programmatically useful indicators (although not sufficient, in themselves, as summary measures of program performance); they point to how adequately services are reaching intended beneficiaries. The same cannot be said for ANC 4+. This indicator has been used as an overall proxy for delivery of a package of needed antenatal care. As demonstrated by the analysis here, it serves this role poorly. For most of the elements of care, there were marked quality–coverage gaps. And high ANC 4+ coverage can be completely compatible with a large quality–coverage gap (for example, see Congo Brazzaville, Indonesia, Namibia, and Swaziland, in Table 1). Furthermore, its widespread use as the single benchmark indicator for antenatal care has the very unwelcome effect of directing the attention of clinicians and program managers toward optimizing the *number* of antenatal visits rather than ensuring delivery of the important *substance* of that care. This effect is exacerbated when attendance at 4 ANC visits is incentivized under conditional cash transfer programs, or when it serves as part of the basis for performance-based financing schemes.

Furthermore, continued use of this indicator reinforces the impression that an abbreviated schedule of antenatal visits is adequate. Recent further analysis^6^ of the original WHO research that gave rise to the 4-visit recommendation has demonstrated a 27% higher risk of fetal death among those randomized to the abbreviated schedule. Moreover, with eclampsia/preeclampsia emerging as the leading cause of maternal death in certain countries, there is renewed recognition of the importance of more vigilant routine screening and timely response to worsening preeclampsia, which cannot be accomplished with only 4 visits over the entire pregnancy. Commenting on the secondary analysis of the WHO antenatal care trial, Justus Hofmeyr^7^ makes the case that:

An increased number of routine visits may detect asymptomatic conditions such as preeclampsia, fetal growth restriction or reduced fetal movements earlier, allowing more timely intervention. The importance of the content and quality of routine antenatal care should not be lost to policy makers when decisions about numbers of visits with the available resources are being made.

Recent analysis found higher risk of fetal death with the abbreviated ANC schedule of visits.

It is time to drop the use of ANC 4+. It does not reliably tell us how adequate ANC services are, and relying on it encourages program managers and clinicians to focus on mere contact rather than on the content of care. Furthermore, as we have noted, 4 visits are not enough.

### Alternative Indicators to Measure ANC Program Performance

ANC 4+ has been retained, to date, as the key global benchmark indicator for antenatal care not because there are passionate defenders of its validity but because there is a perception that there is no readily available alternative. But there is.

In principle, an attractive option would be the **proportion of women who report receiving the full set of specific elements of care** measured. This can be readily determined from survey data. Kyei and colleagues^8^ have done such analysis based on data from the 2007 Zambia DHS, using an overlapping, but not identical, set of ANC-related indicators to those used here.* In their study, “good-quality ANC” was defined as attending at least 4 ANC visits with a skilled provider and receiving at least 8 of the 10 antenatal interventions used in their analysis; “moderate-quality ANC” required 4 visits and 5–7 of the 10 antenatal interventions. In this paper, similar analysis found that in about one-third of the surveys (15 of 41), the proportion of women receiving all 8 services (or fewer, if a particular indicator was not included in the survey) was zero. So the utility of this specific measure is constrained by its lack of discriminating power. A further limitation is that, unlike a simple average across indicators—which can be easily calculated from corresponding indicators already tracked by routine health information systems—a measure of receipt of a full set of services at the level of the individual woman would, for the foreseeable future, only be feasible in periodic population surveys and special studies.

So we propose adopting, as a summary measure of antenatal program performance at the population level, the **simple average of a set of available indicators for receipt of specific services** (such as presented in this paper). For use at the global level, to ensure strict comparability, it may be necessary to restrict this composite indicator to content elements that are common across all countries. This would imply retaining HIV- and malaria-related interventions in the summary measure only for within-country use, in settings where this is warranted by local epidemiology and public health priorities. We propose that the same approach be used for periodic population surveys and for ongoing monitoring using routine health information systems.

An alternative indicator to ANC 4+ to measure program performance could be a simple average of receipt of a set of key antenatal services.

Certainly, the specific components of an average measure merit further debate and discussion. There may be other interventions tracked by health management information systems and measured by DHS or other periodic surveys that could be included (for example, those in the analysis done by Kyei and colleagues^8^). Likewise, average total number of ANC visits could be included in the summary average measure.

Such an average coverage measure would reflect much better how well the needs of the population are actually being met, with regard to the substance of antenatal care, than does the ANC 4+ indicator.

This brings us to an important issue of terminology. Shengelia and colleagues^9^ have provided a formal description of “effective coverage,” which comprises individual-level need, utilization, and quality. Bryce and colleagues^10^ have criticized this concept as unnecessarily complex and not readily measurable.

In the global *child health* sphere, use of the term “coverage” is relatively unproblematic, as it is normally used to refer to delivery of specific technical interventions. However, in global maternal health discourse, “coverage” commonly refers to mere contact (notably ANC 4+ and skilled birth attendance), and these measures are used as proxies for adequate delivery of needed care to a population.

For *maternal health*, a shift toward use of indicators of overall program performance that take account of the actual substance of care provided is certainly called for. For that purpose, we would endorse use of indicators that track “effective coverage,” as the term is used by Kyei and colleagues^8^—“the proportion of the population who need a service that receive it with sufficient quality [for it] to be effective.” In the case of antenatal care, using a more appropriate summary metric for overall program performance, as proposed here, would help effect a much-needed shift in focus, putting the content back into contact.

## Footnotes

* Weight measurement, height measurement, blood pressure measurement, urine sample taken, blood sample taken, voluntary counseling and testing for HIV offered, iron supplementation provided, antimalarial drug provided for intermittent preventive treatment of malaria, birth preparedness plan discussed, and deworming and tetanus toxoid vaccination provided.
